# Determining the Effect of Temperature on the Growth and Reproduction of *Lasioderma serricorne* Using Two-Sex Life Table Analysis

**DOI:** 10.3390/insects12121103

**Published:** 2021-12-10

**Authors:** Tao Wang, Yan-Ling Ren, Tai-An Tian, Zhi-Tao Li, Xing-Ning Wang, Zhi-Yi Wu, Jian Tang, Jian-Feng Liu

**Affiliations:** 1 Guizhou Light Industrial Technical College, Guiyang 550032, China; wangtaotougao@126.com (T.W.); tang123tang456jian@163.com (J.T.); 2 Guizhou Provincial Key Laboratory for Agricultural Pest Management of the Mountainous Region, Institute of Entomology, Scientific Observing and Experimental Station of Crop Pest in Guiyang, Ministry of Agriculture, Guizhou University, Guiyang 550025, China; 3 Guizhou Academy of Forestry, Guiyang 550005, China; taian2015@126.com; 4 China Tobacco Guizhou Import and Export Co., Ltd., Guiyang 550005, China; zhitaolitobacco@sina.com; 5 Guiyang Customs Technology Center, Guiyang 550081, China; wangxingning2004@126.com; 6 Zhejiang Academy of Science & Technology for Inspection & Quarantine, Hangzhou 311202, China; wzy@zaiq.org.cn

**Keywords:** high temperature, cigarette beetle, life history, fecundity, population

## Abstract

**Simple Summary:**

The developmental time of *L. serricorne* significantly decreases as the temperature increases from 21 °C to 33 °C. Adult *L. serricorne* have a relatively longer oviposition period and the highest fecundity at a temperature of 33 °C. *Lasioderma serricorne* has the highest age-stage-specific survival rate (*S_xj_*) at a temperature of 27 °C, but a higher fecundity (*m_x_* and *l_x_m_x_*) is observed at temperatures of 30 °C and 33 °C. A comprehensive analysis shows that among the experimental temperatures, a temperature between 30 °C and 33°C is the most appropriate for the population development of the pest. Our research results provide theoretical information for the control of pests and mass rearing of *L. serricorne*.

**Abstract:**

The cigarette beetle *Lasioderma serricorne* (Fabricius) is a major pest of stored products worldwide, especially tobacco and foods, causing huge economic losses. This study aimed to experimentally investigate the population dynamics of this pest at different temperatures and provide theoretical input for its control. Populations of *L. serricorne* were established under laboratory conditions at five temperatures (21 °C, 24 °C, 27 °C, 30 °C, and 33 °C). Results showed that an increasing temperature significantly affected the developmental time, longevity, oviposition period, and fecundity of *L. serricorne*. Both the longevity and fecundity of adult beetles were significantly reduced as the temperature increased. High temperatures significantly reduced the total duration of the preoviposition period but prolonged the oviposition period of *L. serricorne*. Increasing the temperatures from 21 °C to 33 °C significantly influenced the life table parameters of *L. serricorne*. The intrinsic increase rate (*r*), finite increase rate (*λ*), and gross reproductive rate (*GRR*) all increased with a greater rearing temperature, but mean generation time (*T*) was significantly shortened. To our best knowledge, this is the first report to detail the entire life history of the cigarette beetle in response to different temperatures when reared on tobacco dry leaves. This finding may provide basic information on the occurrence of *L. serricorne* in a warehouse setting and its mass rearing.

## 1. Introduction

The cigarette beetle *Lasioderma serricorne* (Fabricius), (Coleoptera: Anobiidae), is a major worldwide pest of stored products, such as tobacco, tea, Chinese crude drugs, dried vegetable products, dried fruit, and animal substances [1]. The cigarette beetle has four life stages (egg, larva, prepupae and pupae, and adult): the feeding larvae of *L. serricorne* cause most of the damage to the infested commodities, but adults of the beetle also are capable of cutting holes when trying to escape or enter packaged commodities, although inflicting damage to a lesser extent than larvae [2,3]. Notably, the cigarette beetle is an important pest of tobacco and can infest unprocessed tobacco leaves and manufactured tobacco products [1,2]. The adults normally lay eggs on the surface of tobacco leaves, and then the larvae might consume the leaf into the flour; this herbivory severely damages the potential stored product, not only via what is consumed by larvae but also by a reduction in its market value caused by remnant excrement, skins, or dead bodies [1]. Due to its special life habit of completing all life stages inside the stored product, it is very difficult to devise an efficient method to control this beetle in tobacco warehouses. The extent of damage caused by a pest mainly depends on how rapidly the population develops [4].

The dynamics of insect populations are influenced by environmental factors, such as suitable food sources, high humidity, and optimal temperatures [2]. The paramount factor, however, is temperature. Edde (2019) reviewed the developmental time of immature stages and the total developmental period of *L. serricorne* reared at different temperatures (from 25 °C to 32 °C) with feeding on different rearing media [2]. Different developmental stages of *L. serricorne* differ in their response to temperatures. *Lasioderma serricorne* eggs do not hatch at 15 °C or 40 °C [1], and, although larvae could develop under a lower temperature range of 15 °C–19 °C [1,2,5,6], they cannot complete the development process at 40 °C [1]. The optimal temperature range for rapid development of *L. serricorne* under 75% RH is 29–35 °C [7]. The high-temperature threshold of newly hatched larvae on wheatfeed is 37.5 °C [1]. The developmental time of immature stages and the whole immature period is shortened by an increase in temperature [6,8], yet the developmental period is prolonged at 35 °C [8]. Previous studies, however, have only considered the effect of temperature on the growth of *L. serricorne*, but did not focus on its reproduction [6,8,9,10].

Life table analysis is a major tool for investigating the dynamics of insect populations. In particular, an age-stage, two-sex life table can not only reflect the growth, development, reproduction, and survival of female and male insects, but also reveal differences in individual development and overlapping generations in a straightforward manner [11,12]. Establishing robust age-stage, two-sex life tables of pests at different temperatures can accurately predict their occurrence and identify the best timing to control them [13]. Life table parameters of *L. serricorne* have been estimated for different varieties of tobacco, ginger, and turmeric [14,15,16]. However, the performance of *L. serricorne* might show differential responses to various temperatures because of different food types [2]. Although researchers have tested the effects of various temperatures on the duration of immature stages and fecundity of *L. serricorne* on wheatfeed [1] and the length of its life cycle on tobacco [17], there is surprisingly little information on the entire life history of the cigarette beetle at different temperatures on tobacco. Moreover, previous studies found that the mature larvae and pupae of *L. serricorne* are the most suitable hosts for the mass rearing of the ectoparasitoid wasp *Anisopteromalus calandrae* (Howard), and the maximum parasitism of *L. serricorne* on larvae and pupae were 14.14 and 5.19, respectively [18,19]. However, we did not know which temperature was the best culture temperature for the mass rearing of *L. serricorne*. Therefore, in this study, we analyzed the effects of five different temperatures (21 °C, 24 °C, 27 °C, 30 °C, and 33 °C) on the life history of *L. serricorne* by age-stage, two-sex life table theory. The results could provide theoretical input for the occurrence prediction and mass rearing of cigarette beetle.

## 2. Materials and Methods

### 2.1. Insect Material

Larvae of *L. serricorne* were collected on 10 June 2019 from dry tobacco leaves provided by the Guiyang Redrying company of the Guizhou Tobacco Redrying Group (Guiyang, Guizhou, China). They were transferred to a laboratory at the Ecological Food and Agricultural Product Engineering Research Center, Guizhou Light Industry Technical College, where the larvae were reared using dry tobacco leaves in a glass pot in a controlled environment (temperature of 28 ± 1 °C, relative humidity of 75% ± 5%, and a light: dark [L: D] photoperiod of 0 h: 24 h). The colonies were reared for three generations with dry tobacco under laboratory conditions before the experiment was initiated. In order to obtain the same age of eggs, we transferred 10 pairs of *L. serricorne* adults into a glass pot for 24 h, and then these eggs were used in the experiment.

The main instruments used in the experimental investigation included an RXZ-430 constant temperature incubator (Jiangnan Instrument Factory, Ningbo, China) and a Yadu ultrasonic humidifier (Yibo Technology Co., Ltd., Beijing, China).

### 2.2. Effect of Temperatures on The Life History of L. Serricorne 

To test the effect of temperatures on the life history of *L. serricorne*, we set five temperatures, 21 °C, 24 °C, 27 °C, 30 °C, and 33 °C, at a relative humidity of 75% ± 5% under continued darkness. One hundred *L. serricorne* eggs within the same 24 h span were selected for each temperature. Each egg was placed on the same-sized tobacco leaf in a Petri dish (having a diameter of 9 cm and a cover with drilled air holes, with the gap between the dish and cover sealed with tape to prevent the insect from escaping). We observed these samples daily to determine their mortality rate and the time needed to complete every stage of development under different temperatures. The tobacco leaf was replaced before being fully consumed. 

An emerged female adult was paired with a male adult from the same population that emerged in the same period or, if such a male adult was not available, with an additional male adult provided that had been reared under the same conditions. In a given pair, if the male died before the female, that male was immediately replaced. If the female of a pair died before the male, then the recording of the longevity (number of days) and fecundity (number of eggs produced each day) of the female was stopped. A male adult was paired with a female adult from the same population or one reared under the same conditions. Likewise, if the female of a pair died before the male, that female was replaced. If the male died before the female, then the recording of the longevity of that male was stopped. The data collected on pre-oviposition and oviposition periods of *L. serricorne* came from daily observations.

### 2.3. Life Table Analysis

The developmental duration, fecundity, adult longevity, larval survival rate, pupation rate, and egg-to-adult probability of *L. serricorne* were calculated using the collected raw data according to the age-stage, two-sex life table method [20,21]. The age-stage-specific survival rate (*S_xj_*) of a population, that is, the probability that an individual egg develops to age *x* and stage *j*, was calculated using the daily observations of the number of surviving individuals and the number of reproducing individuals in that population.

The age-specific survival rate (*l_x_*) of a population is defined as the rate of hatching eggs that develop and survive to age *x* and is calculated by the following formula:

$$l_{x}=\sum_{j=1}^{m}S_{xj}$$

The age-specific fecundity (*m_x_*) of a population is defined as the average number of eggs produced by the entire population at age *x* and is calculated using this formula:

$$m_{x}=\frac{\sum_{j=1}^{m}S_{xj}f_{xj}}{\sum_{j=1}^{m}S_{xj}}$$

The gross reproductive rate (*GRR*) was calculated as:

$$GRR=\sum^{\;}m_{x}$$

The age-specific reproductive value (*l_x_m_x_*) of a population is defined as the product of its age-specific survival rate (*l_x_*) and age-specific fecundity (*m_x_*) and is calculated using this formula:

$$l_{x}m_{x}=\frac{\sum_{j=1}^{m}S_{xj}f_{xj}}{\sum_{j=1}^{m}S_{xj}}\sum_{j=1}^{m}S_{xj}$$

The age-stage-specific life expectancy (*e_x__j_*) is defined as the number of days that individuals of age *x* and stage *y* can continue to live and is calculated using this formula:

$$e_{xj}=\sum_{i=x}^{n}\sum_{j=y}^{m}S_{ij}$$

where *n* is the last age of individuals in the population, *m* is the number of stages, and *S_ij_* is the probability that individuals of age *x* and stage *y* survive to age *i* and recruit into stage *j*.

The age-stage-specific fecundity (*v_xj_*) measures the contribution of an individual of age *x* and stage *y* to the future of the population and is calculated by this formula:

$$v_{\mathrm{xj}}=\frac{\;e^{-r\left(x+1\right)}}{S_{xy}}\sum_{i=x}^{n}e^{-r\left(x+1\right)}\sum_{j=y}^{m}S_{ij}f_{ij}$$

The intrinsic rate of increase (*r*) of a population, a life table parameter, is calculated using the binary iteration method, as follows:

$$r=\sum_{x=1}^{\infty }(e^{-rx}\sum_{j=1}^{m}S_{ij}f_{ij})$$

The finite rate of increase *λ* is calculated using this formula:

$$\lambda =e^{r}$$

The net reproductive rate (*R*_0_) is defined as the total number of offspring produced by an individual throughout its lifetime and is calculated using this formula:

$$R_{0}=\sum_{x=0}^{\infty }l_{x}f_{x}$$

The mean generation time (*T*) is defined as the time needed by a population that has reached a stable rate of increase (*r* and *λ*) to increase *R*_0_ and is calculated using this formula:

$$T=\frac{lnR_{0}}{r}$$

The demographic raw data in this study were analyzed according to the age-stage, two-sex life table theory [11,20]. The variance and standard errors of developmental duration, fecundity, and longevity of *L. serricorne* under different temperatures were calculated via 100,000 bootstraps; these were performed in the TWOSEX-MSChart computer program [21]. For a proper application of the bootstrap technique, the randomization function was used in TWOSEX-MSChart. Moreover, according to Akca et al. (2015), we used 100,000 resamplings to obtain stable estimates of standard errors. A paired bootstrap test was used to evaluate the differences among developmental duration, fecundity, and longevity of *L. serricorne* under different temperatures (*p* < 0.05). Both bootstrap and paired bootstrap tests were implemented in the TWOSEX-MSChart computer program [21]. SigmaPlot 14.0 software was used to plot the curves for survival rates, developmental times, fecundity, reproductive values, and life expectancy of *L. serricorne*.

## 3. Results

### 3.1. Developmental Time, Survival, Longevity, and Fecundity of L. Serricorne at Different Temperatures

Temperature significantly affected the developmental time, longevity, oviposition period, and fecundity of *L. serricorne* (Table 1 and Table S1). The time needed by *L. serricorne* to complete every developmental stage significantly decreased as the temperature increased from 21 °C to 33 °C. The developmental durations of *L. serricorne* larvae at temperatures of 21 °C, 24 °C, 27 °C, 30 °C, and 33 °C were 71.95, 59.89, 57.42, 35.59, and 33.92 days, respectively, with the developmental duration at 21 °C more than two times that at 33 °C. The longevity of the female and male adults significantly decreased as the temperature increased. The longevity of female adults at 21 °C and 33 °C was 132.59 and 63.57 days, respectively, and that of the male adults was 133.35 and 64.43 days, respectively; hence, the pest’s longevity was nearly two-fold longer at a temperature of 21 °C than at 33 °C. There was, however, no significant difference in the longevity of adults between 30 °C and 33 °C. High temperatures (30 °C and 33 °C) significantly shortened the total preoviposition period but prolonged the oviposition period of *L. serricorne*. Furthermore, the fecundity of female adults peaked (40.49 eggs) at 33 °C, being significantly higher than that at 21 °C, 24 °C or 27 °C.

**Figure 1:**
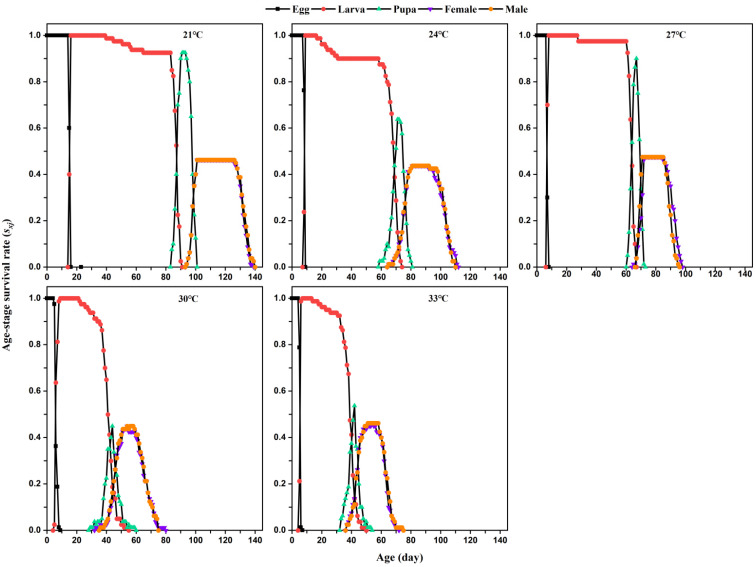
Age-stage-specific survival rates (*S_xj_*) of *L. serricorne* at different temperatures.

**Figure 2:**
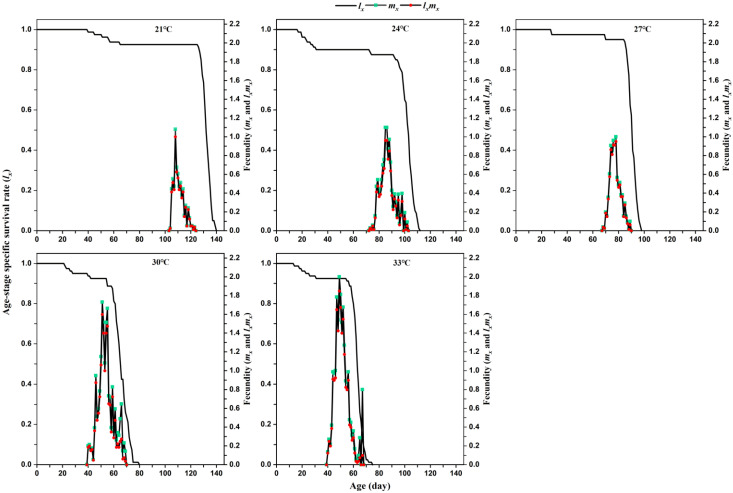
Age-specific survival rate (*l_x_*), age-specific fecundity (*m_x_*), and age-stage-specific reproductive value (*l_x_m_x_*) of *L. serricorne* at different temperatures.

*Lasioderma serricorne* exhibited different age-stage-specific survival rates (*S_xj_*) and age-specific survival rates (*l_x_*) at different temperatures (Figure 1 and Figure 2). The curves indicate the probability that a newly hatched egg survives to age *x* and stage *j*. Overlapping curves indicated the existence of overlapping generations. Temperature significantly reduced the maximum peak *Sxj* of pupae from 21 °C to 33 °C. No differences in the *S_xj_* of females and males were found in each temperature. Compared with the curve trend of *S_xj_* larval stage and *l_x_* at 21 °C, 24 °C, and 27 °C, the curve of *S_xj_* and *l_x_* rapidly declined at 30 °C and 33 °C. However, the curves of age-specific fecundity (*m_x_*) and age-stage-specific reproductive value (*l_x_m_x_*) reached higher peaks at 30 °C and 33 °C than those values at 21 °C, 24 °C, and 27 °C.

**Figure 3:**
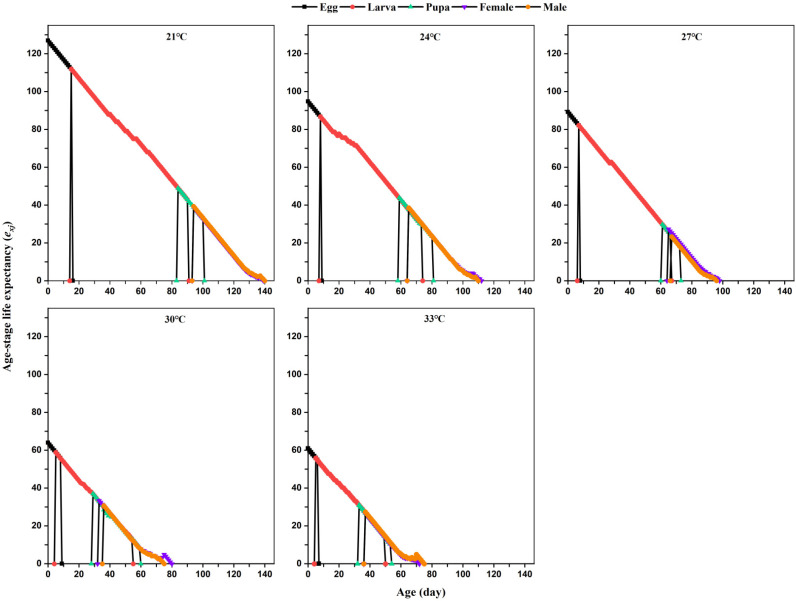
Life expectancies (*e_xj_*) of *L. serricorne* at different temperatures.

Temperature significantly affected the life expectancy *e_xj_* of *L. serricorne* (Figure 3). The life expectancies *e_xj_* of both immature and mature *L. serricorne* decreased as the temperature increased. Notably, the temperature had the greatest impact on the life expectancy of eggs; the life expectancy at 21 °C, 24 °C, 27 °C, 30 °C, and 33 °C was 126.92, 94.74, 89.2, 63.92, and 60.85, respectively.

**Figure 4:**
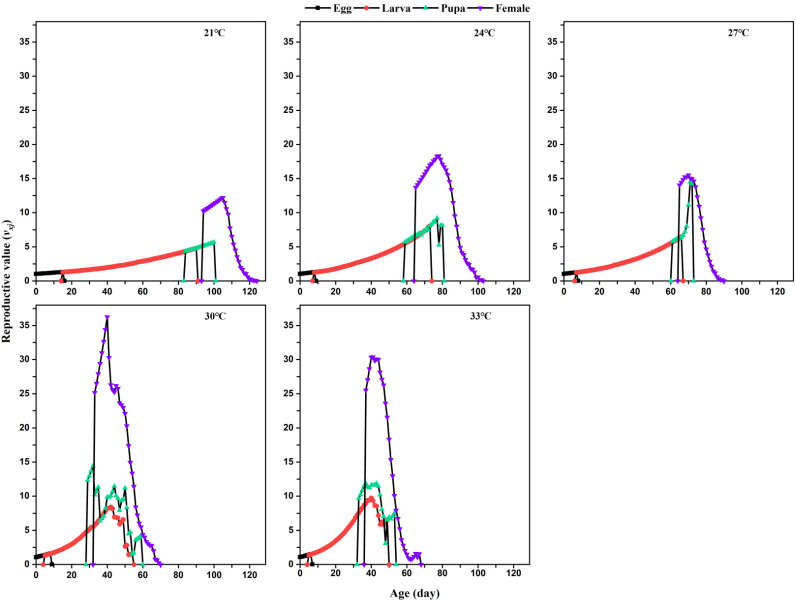
Age-stage specific reproductive value (*v_xj_*) of *L. serricorne* at different temperatures.

The age-stage specific reproductive value (*v_xj_*) of *L. serricorne* at different temperatures differed significantly (Figure 4). The *v_xj_* of *L. serricorne* increased steadily with the temperature increase. The peak *v_xj_* of an *L. serricorne* female occurred earlier at 31 °C (40 days) and 33 °C (41 days) than that female at 21 °C (105 days), 24 °C (78 days), and 27 °C (70 days).

### 3.2. Population Parameters of L. Serricorne at Different Temperatures

The population life table parameters of *L. serricorne* varied significantly as the temperature increased from 21 °C to 33 °C (Table 2 and Table S2). The intrinsic increase rate *r*, finite increase rate *λ*, net reproductive rate *R*_0_, and gross reproductive rate (*GRR*) were all positively enhanced by temperature. The net reproductive rates at temperatures of 21 °C and 33 °C were 6.2 and 18.725, respectively, the latter being more than three times the former value. However, the mean generation time, *T*, of *L. serricorne* shortened as the temperature rose. The mean generation times at 21 °C and 33 °C were 111.373 and 50.737 days, respectively, with the former more than double the latter value.

## 4. Discussion

Being poikilothermic, insects are very sensitive to environmental temperature, which affects their behavior, life cycle (particularly growth and development), and distribution [22]. To date, life table parameters of *L. serricorne* have been studied and obtained for different varieties of food products that this beetle pest infests [14,15,16]. Ours is the first detailed study of how temperatures affect the development and reproduction of *L. serricorne* on dry tobacco leaves. We found that temperature significantly influenced the developmental time, longevity, fecundity, and life table parameters of *L. serricorne* on tobacco. Higher temperatures (30 °C and 33 °C) significantly reduced the development and longevity but increased the fecundity of *L. serricorne* on dry tobacco leaves. Increasing temperatures significantly improved the intrinsic increase rate, finite increase rate, and gross reproductive rate, but shortened the mean generation time of *L. serricorne*. These results are consistent with the previous studies that the optimum temperature for quickly larval development of *L. serricorne* is around 32 °C [1,17].

Temperature influences the developmental time of immature stages of *L. serricorne* [1,2,17]. Raising the temperature from 20 °C to 32.5 °C significantly reduced the developmental time of *L. serricorne*’s larval stage on wheatfeed at 70% RH, but its duration increased at 35 °C [1]. When the temperature was increased from 20 °C to 35 °C, the incubation and pupal period of *L. serricorne* both decreased, whereas at 37.5 °C, they increased. The durations of the eggs, larvae, pupae, and total immature stages of *L. serricorne* were found to decrease with a temperature increase from 20 to 30 °C [6]. In our study, a similar result was obtained. The rearing temperature significantly affected the development of every *L. serricorne* immature stage and its total immature period; its shortest developmental duration for the immature stages was at 33 °C. The beetle’s larval stage duration (71.95 days) at 21 °C on tobacco (this study) was similar to that when fed wheatfeed (69.6 days) at 20 °C; however, the duration of its larval stage on tobacco (this study) at 27 °C (57.42 days), 30 °C (35.59 days), and 33 °C (33.92 days) was two times longer than when reared on wheatfeed at 27.5 °C (22.4 days), 30 °C (18.4 days), and 32.5 °C (15.7 days) [1]. On the contrary, larval development periods in our study were significantly lower than those for the beetle when fed with bread crumbs at 20 °C (187.2 days), 25 °C (75.7 days), 27 °C (51.3 days), and 30 °C (46.5 days) [6]. The discrepancies in the larval duration responses to temperature changes might be caused by the food types used to rear them, as food source identity is known to influence the larval period of *L. serricorne* [1,7,22]. Nonetheless, the pupal period of *L. serricorne* at various temperatures in this study with tobacco showed a similar trend to that for the beetle reared on wheatfeed [1]. Increasing temperatures significantly shorted the longevity of *L. serricorne* females and males, with no significant sex difference found. Similarly, the mean length of adult lifespan fell with an increase in temperature from 20 °C to 35 °C, with no sexually dimorphic response in longevity between females and males induced by the changed temperature [1]. There is a similar decrease in the length of the tobacco beetle’s life cycle as the temperature 24 °C, 28 °C and 32 °C at 45%, 60%, and 75% RH, but a slight increase in its longevity at 36 °C [17].

We found that changing temperature was able to influence the oviposition period and fecundity of *L. serricorne* on tobacco. Higher temperatures could prolong the oviposition period of female beetle from 4.51 days at 21 °C to 7.76 days at 33 °C. In early work, Powell (1931) reported that oviposition continues for 3 to 10 days at 32 °C [17]. By contrast, Howe (1957) reports that the individual female beetle has a longer oviposition period (14 days to 20 days) at 22.5 °C, which is shortened by more than half a (6 days to 9 days) at 35 °C [1]. The higher temperature did not influence the oviposition period in our study in comparison with previous studies [1]. Meanwhile, our study is the first to find that high temperature markedly increased the number of eggs produced per female fed with tobacco from 13.41 at 21 °C to 40.49 at 33 °C. Likewise, Powell (1931) reported a similar number of eggs (43.57) at 32 °C on yeast [17]. However, the fecundity of *L. serricorne* fed with tobacco (34.81 eggs) in this study at 30 °C was significantly lower than the corresponding value for the beetle feeding on dry turmeric (Duggirala [52.57 eggs], Tekurpet [58 eggs], and Mydukur local [50.85 eggs]) [15] and dry ginger (Kerala [53.28 eggs], Local siddipet [57.14 eggs], and Rajitha [50.85 eggs]) [14].

Temperature significantly influenced the life table parameters of *L. serricorne*. In this study, higher temperature significantly increased the intrinsic increase rate (*r*), finite increase rate (*λ*), and gross reproductive rate (*GRR*), but there was no significant difference in those population-level responses between 30 °C and 33 °C. The intrinsic increase rate of *L. serricorne* fed with tobacco types (flue-cured, burley, and cigar wrapper) at 27 °C is 0.045, 0.0306, and 0.0286, respectively, which is lower than the intrinsic increase rate (0.08) in our study at the same temperature [16]; a similar pattern was also found for the mean generation time. Nevertheless, in our study, the finite increase rate (1.09) of *L. serricorne* was similar to that reported for the beetle when fed flue-cured (1.0454), burley (1.0308), and cigar wrapper (1.028) [16].

In conclusion, this study demonstrated that an increase in temperature from 21 °C to 33 °C could significantly affect the developmental time, longevity, fecundity, and life table parameters of *L. serricorne* on tobacco. Previous research has mainly focused on the effect of temperature on the growth and development of *L. serricorne*, but this is the first report to examine the entire life history of the cigarette beetle on showing how it responds to different temperatures on tobacco. Based on the shorter developmental time and higher fecundity of *L. serricorne*, the best cultural temperature for the mass rearing of *L. serricorne* was 33°C. Our results provide basic information that could prove useful in predicting the occurrence of *L. serricorne* under a constant temperature and the mass rearing of *L. serricorne*. However, in this study we did not evaluate the fitness of *A. calandrae* parasitism on *L. serricorne* rearing at different temperatures. Therefore, we hope our findings could spur future research to understand the performance of the parasite *A. calandrae* on *L. serricorne* under different temperatures.

## Figures and Tables

**Table 1:** Developmental time and reproduction parameters of *L. serricorne* at different temperatures.

Population Parameter	N	21 °C	N	24 °C	N	27 °C	N	30 °C	N	33 °C
Developmental time of egg (days)	80	15.6 ± 0.06 a	80	8.76 ± 0.05 b	80	7.3 ± 0.05 c	80	6.54 ± 0.1 d	80	5.8 ± 0.05 e
Developmental time of larva (days)	74	71.95 ± 0.21 a	72	59.94 ± 0.37 b	78	56.87 ± 0.17 c	76	35.33 ± 0.49 d	74	34 ± 0.38 e
Developmental time of pupa (days)	74	10.7 ± 0.05 a	70	6.26 ± 0.07 b	76	5.62 ± 0.08 c	74	4.05 ± 0.04 d	74	4.00 ± 0.00 d
Adult longevity (days)	74	34.7 ± 0.27 a	70	28.5 ± 0.35 b	76	21.57 ± 0.3 c	74	20.65 ± 0.35 d	74	19.86 ± 0.29 d
Total longevity of female individuals (days)	37	132.59 ± 0.5 a	35	103.26 ± 0.82 b	38	92.32 ± 0.54 c	37	66.51 ± 0.92 d	37	63.57 ± 0.58 e
Total Longevity of male individuals (days)	37	133.35 ± 0.55 a	35	103.63 ± 0.63 b	38	90.32 ± 0.42 c	37	66.81 ± 0.81 d	37	64.43 ± 0.57 e
Total preoviposition period (TPOP) (days)	37	107.81 ± 0.43 a	35	82.09 ± 0.61 b	38	74.68 ± 0.42 c	37	50.41 ± 0.77 d	37	47.38 ± 0.63 e
Adult preoviposition period (APOP) (days)	37	9.54 ± 0.35 a	35	7.14 ± 0.22 b	38	4.42 ± 0.27 c	37	4.22 ± 0.22 c	37	3.68 ± 0.13 d
Oviposition days (*O_d_*)	37	4.51 ± 0.35 c	35	7.46 ± 0.46 a	38	5.76 ± 0.29 b	37	7.27 ± 0.39 a	37	7.76 ± 0.4 a
Fecundity (no. of eggs)	37	13.41 ± 1.23 c	35	23.29 ± 2.36 b	38	18.29 ± 1.97 b	37	34.81 ± 3.05 a	37	40.49 ± 3.78 a

A paired bootstrap test was used to detect a significant difference in developmental time and reproduction parameters of *L. serricorne* at five different temperatures. Means within a row followed by the same letter are not significantly different. Standard errors were estimated by resampling (100,000 bootstrap samples).

**Table 2:** Life table parameters of *L. serricorne* populations reared at different temperatures.

Rearing Temperature	Intrinsic Increase Rate *R* (Days^−1^)	Finite Increase *Rate* *Λ* (Days^−1^)	Net Reproductive Rate *R*_0_	Gross Reproductive Rate *GRR*	Mean Generation Time *T* (Days)
21 °C	0.0164 ± 0.0014 c	1.0165 ± 0.0014 c	6.2 ± 0.935 c	6.7 ± 0.989 c	111.373 ± 0.49 a
24 °C	0.027 ± 0.002 b	1.027 ± 0.002 b	10.187 ± 1.64 b	11.83 ± 1.861 b	87.066 ± 0.826 b
27 °C	0.0276 ± 0.002 b	1.028 ± 0.002 b	8.387 ± 1.385 bc	9.2 ± 1.444b c	78.199 ± 0.58 c
30 °C	0.0521 ± 0.003 a	1.053 ± 0.003 a	16.1 ± 2.391 a	18.88 ± 2.764 a	53.325 ± 0.915 d
33 °C	0.0577 ± 0.003 a	1.059 ± 0.003 a	18.725 ± 2.848 a	21.41 ± 3.218 a	50.737 ± 0.622 e

A paired bootstrap test was used to detect significant difference in developmental time and reproduction parameters of *L. serricorne* in different temperatures. Means within a row followed by the same letter were not significantly different. Standard errors were estimated by resampling (10,000 bootstrap samples).

## Data Availability

None.

## Supplementary Materials

The following are available online at https://www.mdpi.com/article/10.3390/insects12121103/s1, Table S1: P value of population parameters of *L. serricorne* detected a paired bootstrap test between two temperatures, Table S2: P value of population parameters of *L. serricorne* detected a paired bootstrap test between two temperatures.
