# Supplementary material for: Determining the Effect of Temperature on the Growth and Reproduction of Lasioderma serricorne Using Two-Sex Life Table Analysis
Source: Insects. 2021 Dec 10;12(12):1103. doi: 10.3390/insects12121103 (PMC8708634; doi:10.3390/insects12121103)
Supplement: Supplementary file 1 [file insects-12-01103-s001.zip › insects-1361109-supplementary.pdf]

Table S1. P value of population parameters of *L. serricorne* detected a paired bootstrap test between two temperatures

**Population parameter**	**P (21/24℃)**	**P (21/27℃)**	**P (21/30℃)**	**P (21/33℃)**	**P (24/27℃)**	**P (24/30℃)**	**P (24/33℃)**	**P (27/30℃)**	**P (27/33℃)**	**P (30/33℃)**
Developmental time of egg (days)	0	0	0	0	0	0	0	0	0	0
Developmental time of larva (days)	0	0	0	0	0	0	0	0	0	0.03
Developmental time of pupa (days)	0	0	0	0	0	0	0	0	0	0.23
Adult longevity (days)	0	0	0	0	0	0	0	0	0	0.00033
Total longevity of female individuals (days)	0	0	0	0	0	0	0	0	0	0.00665
Total Longevity of male individuals (days)	0	0	0	0	0	0	0	0	0	0.015720
Total preoviposition period (TPOP) (days)	0	0	0	0	0	0	0	0	0	0.00248
Adult preoviposition period (APOP) (days)	0	0	0	0	0	0	0	0.54849	0.01214	0.03338
Oviposition days (O_d)	0	0.00535	0	0	0.00196	0.75312	0.61484	0.00205	0.00002	0.37408
Fecundity (no. of eggs)	0.00029	0.03407	0	0	0.1005	0.00298	0.00016	0.00002	0.00001	0.23512

Table S2. P value of population parameters of *L. serricorne* detected a paired bootstrap test between two temperatures

**Population parameter**	P (21/24℃)	P (21/27℃)	P (21/30℃)	P (21/33℃)	P (24/27℃)	P (24/30℃)	P (24/33℃)	P (27/30℃)	P (27/33℃)	P (30/33℃)
**Intrinsic increase rate *r* (days -1)**	0.00003	0.00005	0	0	0.72429	0	0	0	0	0.19481
**Finite increase rate *λ* (days -1)**	0.00003	0.00005	0	0	0.72426	0	0	0	0	0.19486
**Net reproductive rate R_0**	0.03418	0.13395	0.00011	0.00004	0.48643	0.04122	0.00889	0.00754	0.00137	0.47725
**Gross reproductive rate *GRR***	0.01457	0.14976	0.00004	0.00002	0.26461	0.03426	0.00928	0.00191	0.00055	0.5434
**Mean generation time *T* (days)**	0	0	0	0	0	0	0	0	0	0.01815
